# Analysis of peri-implant bone defects by using cone beam computed tomography (CBCT): an integrative review

**DOI:** 10.1007/s11282-023-00683-w

**Published:** 2023-04-14

**Authors:** J. A. Costa, J. M. Mendes, F. Salazar, J. J. Pacheco, P. Rompante, M. I. Câmara

**Affiliations:** 1 Medicine and Oral Surgery Department, University Institute of Health Sciences (IUCS), CESPU, 4585-116 Gandra, Portugal; 2 UNIPRO—Oral Pathology and Rehabilitation Research Unit, University Institute of Health Sciences (IUCS), CESPU, 4585-116 Gandra, Portugal

**Keywords:** CBCT, Cone-beam computed tomography, Dental implant, Peri-implant, Bone loss

## Abstract

The objective of this work was to perform an integrative review of the inspection of peri-implant bone defects using cone beam computed tomography (CBCT). An electronic search was performed in the PubMed database using the following scientific terms: CBCT or Cone Beam computed tomography; dental implant; peri-implant; bone loss; defects. The survey identified 267 studies, of which 18 were considered relevant to this study. These studies provided important data taking into account the accuracy of cone beam computed tomography in the detection and measurement of peri-implant bone defects such as fenestrations, dehiscence and intraosseous circumferential defects. The effectiveness of CBCT in aiding in geometric bone calculations and in the diagnosis of peri-implant defects was influenced by factors such as artefacts, defect size, bone wall thickness, implant material, adjustment of acquisition parameters and observer experience. A not insignificant number of studies compared intraoral radiography to CBCT in the detection of peri-implant bone loss. CBCT was clearly superior to intraoral radiography in the detection of all peri-implant bone defects, except for defects located in the interproximal zone. In general, studies have shown that peri-implant bone measurements adjacent to the implant surface can be correctly determined, as well as the diagnosis of peri-implant bone defects with an average discrepancy of less than 1 mm from the actual measurement of the defect.

## Introduction

The importance of radiology in assessing the bone around the implants is undeniable. In this sense, postoperative radiographic evaluation of dental implants and the advantages and disadvantages of different radiographic modalities are important [1].

In 1998/1999, Mozzo et al. introduced cone beam computed tomography (CBCT) (NewTom®, Verona, Italy), which, depending on the specific configurations of the device and the CBCT units, allows three-dimensional images to be obtained with relatively low doses of radiation [2].

The detection of peri-implant bone loss is considered an important criterion for assessing implant performance and diagnosing potential peri-implantitis [2, 3]. Current guidelines recommend intraoral radiographs (peri-apical radiography) when the peri-implant clinical evaluation indicates disease [1, 4–10]. Intraoral radiography has been widely used to assess changes around implants, mainly due to its considerable advantages, such as low cost, immediate availability, good patient tolerance, ease of use and the ability to provide high resolution images for accurate measurements in implant sites [11]. However, limitations such as geometric distortions, anatomical overlap and inability to represent oral bone levels result in this technique having low sensitivity in detecting early bone loss [7, 12, 13]. These limitations can be problematic in some cases, as the initial bone loss usually occurs in the buccolingual area of an implant due to the relative lack of bone thickness in this area [1].

In cases where three-dimensional visualization of the bone is necessary, CBCT may be an alternative [7, 9, 14, 15]. In implantology, CBCT has been used mainly in the evaluation of surgical sites before the insertion of the implant or of postsurgical complications [4, 7], allowing the precise and early detection of peri-implant bone loss morphology and therefore assisting in the selection of the most appropriate treatment and improving the overall prognosis of patients with compromised implants [4, 15].

The benefits of CBCT include its distortion-free and overlay-free images; however, the relatively high radiation exposure and artefacts in the vicinity of metallic objects limit the application of this technique as a method for routine evaluation of dental implants [1, 9, 10]. These artefacts can alter linear measurements in CBCT images and significantly decrease image quality. In addition, image quality can be negatively influenced by factors related to the patient, such as movement image artefacts [16]. The performance of CBCT as a method for diagnosing peri-implant bone loss is still unclear.

The purpose of this work was to perform an integrative review of the use of CBCT for peri-implant bone loss identification and measurement. It was hypothesized that CBCT would show greater precision in the detection of peri-implant bone defects than traditional X-ray techniques.

## Methodology of bibliographic research

A bibliographic search was carried out in the PubMed database (via the National Library of Medicine) using the following combination of search terms: “CBCT” or “Cone beam computed tomography” AND “dental implant” AND “peri-implant” AND “bone loss” AND “Defects”.

The studies were considered eligible if they addressed the question of the focus, “What is the diagnostic value of CBCT images in direct peri-implant bone measurements?”

Inclusion criteria included articles published in the English language, in vivo and in vitro studies, meta-analyses or bibliographic reviews that diagnosed peri-implant bone loss using CBCT, studies that analysed the accuracy of CBCT in peri-implant bone analysis and studies comparing intraoperative or histological reference measures showing measurable results of diagnostic performance. Additionally, studies that analysed the modifying factors of the performance of CBCT in peri-implant bone analysis were also included.

The exclusion criteria included articles that did not answer the focus question.

The total number of articles was compiled for each combination of key terms; subsequently, duplications were removed using Mendeley® (London, UK) citation manager (version 1.19.4).

A preliminary assessment of the abstracts was carried out to determine whether the articles corresponded to the objective of the study. The selected articles were read and evaluated individually regarding their purpose and relevance.

**Fig. 1:**
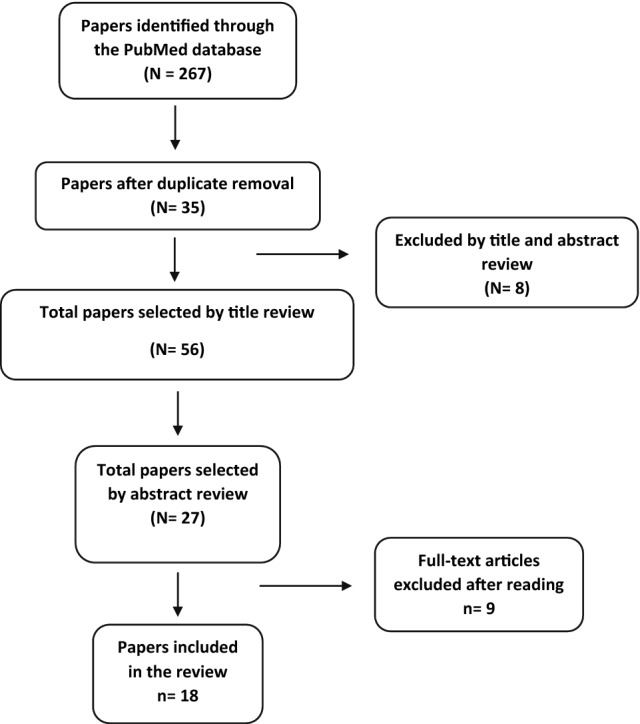
Preferred Reporting Items for Systematic Reviews and Meta-Analysis (PRISMA) flow diagram for the search strategy

The PRISMA flowchart describing the study selection process is shown in Fig. 1.

For this review, the following factors were considered: author, title, year of publication, type of study (clinical, in vivo or in vitro study), number of implants analysed, type of reference or comparative measure used in the CBCT and other X-ray diagnostic means (panoramic or intraoral X-ray), and reported results.

## Results

The literature search identified a total of 267 articles on PubMed using keyword combinations. A total of 56 articles were selected by title, and after eliminating duplicates with the citation manager Mendeley, 35 articles were considered.

Abstracts were read, and 8 articles were excluded because they did not meet the inclusion criteria. The remaining potentially relevant studies were evaluated.

Of these studies, nine were excluded because they did not provide comprehensive data regarding the objective of the present study. Thus, 18 studies were included in this review.

In total, 14 in vitro studies, 3 in vivo and 1 systematic review were included.

The main characteristics and conclusions of the study are summarized in Table 1.

**Table 1:** Relevant data from selected studies

Autor	Study model and number of implants used	Analised factor	Reference or comparative measure	Results
Schriber et al. [16]	In vitro Angle of the mandible of six fresh defrosted pig jaws Implants 12 (6 test group) Ti, ti-zr or zro2	Buccal peri-implant bone defects was created as a dehiscence: 3 mm width 5 mm height 1/2 mm depth (with abutment)	Macroscopic defect measurement	The linear measurements of peri-implant defect were underestimated by < 1 mm on average CBCT showed high diagnostic accuracy for peri-implant b one defect detection Low dose protocols could be a promising imaging modality
George Pelekos et al. [3]	In vitro Porcine rib bone Implants 36 (12 implants for each type of defect)	4wall defect with buccal dehiscence (diameter 6,5 mm) (depth 6 mm) 3wall circumferential defect With buccal Dehiscence (depth 4 mm) buccal dehiscence (depth 4 mm)	Macroscopic defect measurement Fourteen masked examiners evaluated 324 pas and 108 cbct images	Discrepancy to real value < 1 mm The overall diagnostic accuracy of cbct was high (> 96%) for all types of defects Cone beam computed tomography showed better diagnostic accuracy in the detection of peri-implant defects that pa
Schwindling et al. [13]	In vitro Bovine ribs Implants 24 titanium (with crown)	Width and depth 1–2 mm 4 wall 3 wall 2 wall 1 wall Acid conditioning of defects	Macroscopic defect measurement	Ld-cbct provides additional information regarding the geometry of defects than pa No significant difference was found between the two cbct techniques: high dose and low dose The following order was found for classification of the different defect types (sensitivity/specificity): hd-cbct (0.96/0.99) > ld-cbct (0.93/0.98) > ir (0.71/0.95)
Hilgenfeld et al. [5]	In vitro Bovine ribs Implants 48 zirconia	24 standardized defects (1, 2, 3, 4 wall) of 1 mm and 3 mm Acid conditioning of defects	-macroscopic defect measurement	Less sensitivity for 1-mm defects than for 3-mm defects High sensitivity for correct detection of defects type for cbct (0.81). Lowest sensitivity for ir (0.68) Ir can be recommended as the initial imaging method for evaluating peri-implant bone defects at zirconia implants
Steiger-Ronay et al. [6]	In vitro In dental stone 18 titanium implants 18 zirconium dioxide implants	Defect groups: A-no peri-implant defect, B-1 mm width defect C-1.5 mm width defect Measurement of interproximal peri-implant bone defects	Macroscopic defect measurement (digital caliper)	Measurements in cbct always led to an overestimation of the defect width, reaching clinical relevance for zro2 implants Values (mm) for cbct: Titanium A 0.10 ± 0.11, B 0.26 ± 0.05, c 0.24 ± 0.08 Zirconium dioxide a 1.07 ± 0.06, b 0.64 ± 0.37, c 0.54 ± 0.17 Except for ti with defect a, measurements in pr were significantly more accurate in comparison to cbct (*p* ≤ 0.05)
Pinheiro et al. [7]	In vitro Bovin ribs Implants 80	Circumferencial-intrabony defects small: 3–4 mm deep, < 1 mm wide large: 5–6 mm deep, 1–2 mm wide Acid conditioning of defects	Macroscopic defect measurement (periodontal probe)	Cbct imaging at 90 kvp was associated with a significantly higher rate of detection of both small and large chemically simulated bone defects than pa or cbct at 75 kvp. Cbct imaging at 75 kvp proved better than pa for the detection of small defects
González-Martín et al. [14]	In vitro Implants 60	Surgically created dehiscence and fenestration defects	Macroscopic defect measurement (digital calipers)	All devices underestimated bone dimensions although differences among them were not significant Low accuracy in diagnosing peri-implant buccal bone. Accuracy was significantly influenced by buccal bone thickness, especially if < 1 mm, and in presence of peri-implant marginal defects Buccal bone ranged from 0.1 to 2.75 mm in thickness and was not visible in 68%, 63% and 60% of cases when using ct, i-cat and newtom, respectively
Pinheiro, Scarfe et al. [15]	In vitro Bovine ribs Implants 80	Circumferencial-intrabony defects: small: 3–4 mm deep < 1 mm wide Large: 5–6 mm deep 1–2 mm wide Acid conditioning of defects	Macroscopic defect measurement (periodontal probe)	Optimal detection of both chemically simulated peri circumferential implant crestal bone defects is achieved at the least radiation detriment using the smallest fov, the highest number of acquisition frames, and the smallest voxel
Lutz Ritter et al. [12]	Histologic (in vivo) Dogs jaws 26 titanium Implants	Implants were placed in dog jaws with chronic type vestibular defects	Histomorphometry	Mesial bone level (mbl) and distal bone level (dbl) were underestimated by both cr and cbct Cbct overestimated vestibular bone levels but underestimated oral bone levels Vestibular and oral bone thicknesses were overestimated
Kamburoglu et al. [8]	In vitro Human mandible (in human cadaver) 69 implants	Created on the buccal aspect of the marginal bone Dehiscence width and/or depth: Small: 1–3 mm Medium: 3–5 mm Large: > 5 mm	Macroscopic defect measurement (digital callipers)	Low sensitivity for detection of small dehiscence, and good for large dehiscence defects Good specificity For detection of all dehiscence defects No significant difference between 3 fov sizes ( each with voxel size < 0.2 mm) Depth, width and volume measurements of the defects from various cbct images correlated highly with physical measurements Significant correlations were found between physical and cbct measurements (*p* < 0.001)
Kamburoglu et al. [9]	In vitro Edentulous mandibles (human cadaver) Implants 42	Dehiscence defects: 3–4 mm deep 3–4 mm wide	Macroscopic defect measurement (digital callipers)	Poor diagnostic accuracy of cbct for dehiscence detection Metal artefact reduction mode did not improve diagnostic accuracy
Corpas et al. [11]	Histologic (in vivo) In 10 minipigs (jaw) Implants 80	Marginal peri-implant circumferencial-intrabony defects formed during implant surgery	Histomorphometry	Cbct and io images deviate, respectively, 1.20 and 1.17 mm from the histology regarding bone defects Moderate correlation of cbct and pr with histological defect depth
Pinheiro et al. [15]	In vitro Bovine ribs Implants 80	Circumferential- intrabony defects T1 = small: 3–4 mm deep, < 1 mm wide T2 = large: 5–6 mm deep, 1–2 mm wide Acid conditioning of defects	Macroscopic defect measurement (periodontal probe)	Fair diagnostic precision for small intrabony defects detection Good precision for detecting large intrabony defects Significant effects for observer type No significant effects for fov settings on diagnostic precision
Fienitz et al. [2]	Histologic (in vivo) Mandible of foxhound dogs (animal model) Implants 24	Implants placed into chronic, surgically created, boxlike intrabony defects: Wide: 6 mm md/6 mm vl Height: 1–8 mm	Histomorphometry	A minimum buccal bw of 0.5 mm was necessary for the detection of bone in radiology Cbct overestimated defects depth Greater deviation of cbct from histologic measurement was noted at defect with buccal bone thickness of < 0.5 mm than that at > 0.5 mm
Vadiati Saberi et al. [1]	In vitro Bovine ribs Implants 40	10 angular defects (2–3 wall) (3 mm) 10 rectangular fenestration defects (10 mm depth) 10 dehiscence defects (3 mm apically from de crest) 10 defect control	Macroscopic defect measurement	The presence and type of all defects were correctly diagnosed using cbct In the identification of dehiscence defects, cbct showed the highest sensitivity Cbct and opa showed similar auc and sensitivity in the detection of fenestration defects
Eskandarloo et al. [18]	In vitro Bovine ribs Implants 31	Peri-implant fenestration (apical third region of implant)	Macroscopic defect measurement	Newtom had the highest sensitivity (75.81%) and specificity (100%) for detection of fenestration, however the differences are not significant
Liedke et al. [10]	In vitro Pig jaw (bone blocks) Implants 6	Buccal bone conspicuity	Macroscopic measurement	The thinner the buccal bone, the higher the risk that the condition of the buccal bone could not be detected. The use of lower resolution protocols increased the risk that buccal bone was not properly detected (OR Sc = 1.46, OR Cr = 2.00). For both CBCT units, increasing the image reconstruction thickness increased the conspicuity of buccal bone (OR Sc = 0.33, OR Cr = 0.31)
Vanderstuyft et al. [20]	Ex-vivo Human cadaver heads Implants 44	Buccal bone thickness	Macroscopic defect measurement	Due to an average blooming (artificial increase of implant diameter) percentage of 12–15%, the buccal peri‐implant bone thickness was underestimated by 0.3 mm on both CBCT devices. Immediately adjacent to the implant blooming area, a doubtful zone of about 0.45 mm was observed in which the buccal bone was not always visible. Buccal bone that was thick enough to fall outside this doubtful zone could always be visualized

Of the 18 selected studies, 5 in vitro studies evaluated the effectiveness of cone beam computed tomography in detecting peri-implant dehiscence, 3 in vitro studies validated the accuracy of CBCT in the diagnosis of fenestration defects, and 8 studies, 5 in vitro and 3 in vivo, evaluated the performance of CBCT in the detection of peri-implant circumferential intraosseous defects.

A systematic review was also included that evaluated the performance of cone beam computed tomography in the evaluation of peri-implant bone loss.

While the aforementioned studies validated the effectiveness of CBCT in detecting peri-implant defects, 1 in vitro study evaluated the factors that affect the possibility of analysing the bone condition around dental implants using cone beam computed tomography.

Of these studies, 1 in vitro study evaluated the effectiveness of low radiation dose protocols in detecting peri-implant bone defects, while two in vitro studies also validated the effect of the field of view (FOV) and the number of CBCT acquisition frames in detecting chemically simulated bone loss around the implants.

Regarding the method of creating peri-implant defects in vitro, surgical creation was the most common method, while five studies applied a chemical attack with perchloric acid. Two studies placed implants in surgically created chronic alveolar defects [2, 12], and one study analysed defects similar to peri-implant craters formed during the preparation of the implant bed [11].

Regarding the reference standards, all in vitro studies used direct measurements of the defects as a reference, while the three in vivo studies used histomorphometry as the “gold standard”.

The main results are presented below:

- The in vivo studies revealed a high precision of the peri-implant bone measurements, giving a high diagnostic value to CBCT in the detection of peri-implant bone loss, despite the presence of underestimation and overestimation of measurements with respect to histology [2, 11].
- CBCT demonstrated high precision in the diagnosis of peri-implant defects in almost all included studies, with a discrepancy in the real value of the defect averaging less than 1 mm [1, 3, 7, 8, 15–17].
- The main limitation of CBCT is the presence of metallic artefacts, that is, image flaws unrelated to the digitized object, caused by metals such as dental implants and amalgam restorations, or to a lesser extent, by root canal filling materials in endodontic treatments [9].
- Overall, the comparison between CBCT and two-dimensional radiography reported significantly higher sensitivity and specificity values for CBCT, as it was more effective than two-dimensional radiography in detecting and classifying peri-implant bone defects [5, 13].
- Adjustment of technical acquisition parameters in relation to image quality control and exposure to reduced artefacts (for example, mA and kVP) may have a direct impact on detecting relatively small deficiencies in the implant region [15].

## Discussion

The accuracy of CBCT in the analysis of peri-implant bone was tested in different experimental models. In general, the selected studies varied markedly in their design, acquisition parameters of CBCT, image processing and reported diagnostic results, so all data presented should be interpreted with caution, as several factors limit the translatability of these preclinical findings.

### Underestimation and overestimation of peri-implant measurements

#### Animal studies

The in vivo studies revealed a high precision of peri-implant bone measurements, indicating that CBCT had high diagnostic value in the detection of peri-implant bone loss, despite the presence of underestimation and overestimation of measurements when compared with histology [2, 11]. The underestimation or overestimation of the defects depended mainly on the bone thickness of the defect, the implant artefacts and the study design. In the study by Fienitz et al., inaccurate values were reported for peri-implant defects that had an oral bone wall less than 0.5 mm compared to defects greater than 0.5 mm. In this group, the depth of the defects was overestimated by almost 2 mm from the actual depth of the defect, with a maximum overestimation of 5.5 mm. In terms of defects with a peri-implant buccal wall greater than 0.5 mm, significantly greater precision was achieved, resulting in smaller differences in discrepancy, on average 0.7 mm, between radiological and histological evaluation. These results reflect the importance of the study by González-Martín et al. and by Liedke et al., who demonstrated that peri-implant oral bone thickness has a significant influence on radiographic visibility [10, 14]. In the study by Liedke et al., the thinner vestibular bone (less than 1 mm) led to an increased risk that the CBCT image of this zone was classified as "undetectable" compared to a vestibular bone with a thickness greater than 1 mm [10], and González-Martín et al. concluded that with 0.5 mm of buccal bone wall, the probability of its detection was less than 20%, while with a thickness of 2.75 mm, it was identified in 100% of the cases, as for every mm increase in bone thickness, the chances of bone identification increased by 30.6% [14]. Moreover, Corpas et al*.* showed statistically significant correlations between radiographic images obtained with CBCT and histological sections; however, the CBCT images produced, unlike the findings of Fienitz et al., a mean underestimation of the depth of bone defects of 1.2 mm, as 50% of the deviations were less than 0.5 mm and, therefore, considered clinically insignificant [2, 5]. The discrepancy between studies may be due to the design of the histological study [12] because the difficult correspondence between the histological section and the radiographic section for comparison between radiographic measurements and precise histological measurements is a limiting factor.

This could be the first explanation of the difference, albeit minimal, between CBCT and histological measurements. In addition, the sample orientation for CBCT images appears to affect the accuracy of peri-implant bone measurements [12].

In the study by Ritter et al., the orientation of the sample affected the direction of the artefacts, changing the precision of peri-implant measurements; more specifically, the direction of the artefacts, diagonal to the implant axis, artificially increased the bone level on the buccal side by 0.3 mm on average, while the level of the lingual bone was hidden by the extinction of artefacts, resulting in a real underestimation of the bone by approximately 0.83 mm. Measurements in the mesial and distal directions showed a similar effect: in the mesial direction, the beam hardening artefacts attenuated the measured values more strongly with respect to histomorphometry than at the distal sites. Based on these studies, the orientation of the implants in relation to the X-ray axis must be considered in the interpretation of the images because they can directly influence the peri-implant bone measurements. In addition, the importance of adequate bone thickness for efficient peri-implant radiographic identification seems evident.

#### In vitro studies

CBCT demonstrated high precision in the diagnosis of peri-implant defects in almost all included studies, with a discrepancy in the real value of the defect averaging less than 1 mm [1, 3, 7, 8, 15–17]. The factors that determined the accuracy of CBCT in the measurement of bone defects in vitro were mainly the size of the defects and the presence of artefacts [1, 5, 8, 9, 15].

Kamburoglu et al. surgically created dehiscence defects of different sizes in implants inserted in the mandibles of cadavers, classifying the defects according to depth and width as small, between 1 and 3 mm, medium, between 3 and 5 mm, and or large, greater than 5 mm, establishing a high correlation between the depth and width measurements of the defects with the actual physical measurements, although the small defects resulted in less accurate diagnoses than the medium and large defects [8]. Similar results were obtained in the studies by Hilgenfeld et al. and Pinheiro et al., where larger bone defects were generally detected and measured more accurately when compared to smaller ones and, with a progressive increase in the size of the defects, there was a greater capacity for its detection [5, 7].

There are different types and brands of dental implants on the market, each with their own characteristics, and after a careful analysis of the articles, the type of implant material analysed also seemed to influence CBCT measurements in a different way. Titanium has been established as the preferred metal for dental implants due to its unique mechanical properties and its high resistance to corrosion, while zirconium dioxide has become more popular as a biocompatible and aesthetic alternative to titanium due to its biophysical properties and white colour [6]. Schriber et al. evaluated the effectiveness of CBCT in diagnosing peri-implant dehiscence by comparing three dental implant materials: titanium, titanium-zirconium alloy and zirconium dioxide [16]. Although CBCT demonstrated high precision in detecting peri-implant bone defects, linear measurements of the height and width of peri-implant defects were underestimated by an average of < 1 mm. In terms of implant material, ZrO2 had a greater impact in relation to artefacts, resulting in a greater underestimation of the width of peri-implant defects than with other materials (mean error of − 1.28 mm) [16], and Steiger-Ronay et al*.* reported that the deviations of the measurements in relation to the real value of the defects were greater around the zirconium implants than the titanium implants [6].

In some studies, it was suggested that there is also a possible impact of different levels of examiner experience on the interpretation of radiographic images [7, 15]. In the study by LR Pinheiro et al., the ability to diagnose bone defects via CBCT was good for two oral and maxillofacial radiologists with extensive experience in interpreting CBCT images but not very good for an oral and maxillofacial surgeon with extensive experience in planning dental implants [15].

Analysing the results of the studies, the diagnostic value of CBCT for peri-implant defects appears to be high, with minimal discrepancy from the reference values.

Clearly, the transfer to clinical application requires further investigation due to the several disadvantages that an experimental model imparts [4, 15], as in many studies, all soft tissues are removed or are not reproduced, and this can increase radiographic details and contrast [15]. Clinical situations are subject to additional factors, such as movement artefacts, which appear as double margins around a structure, increasing progressively with a longer scan time, which can last up to 20–30 s [9]. Moreover, models of bovine bone ribs are commonly used in in vitro studies because they are similar in contour and dimensions to the human jaw and possess cortical and spongy bone [13]; however, most defects are created surgically, which results in a better defined defect margin than in clinical conditions, where defect margins are generally more diffuse and less well defined [4], although conditioning with 17% perchloric acid allows for a more realistic defect morphology [15].

Therefore, the expected accuracy in real-life situations may be different from that found in studies.

### Comparison with two-dimensional radiography

The importance of radiography in the analysis of the bone condition around the implants is indisputable [1] due to the nonsignificant number of articles comparing CBCT and 2D radiography with the aim of evaluating the most appropriate technique for detecting and measuring peri-implant bone defects. In real clinical situations, the morphology of peri-implant bone defects varies; however, for study purposes, they are classified into three types of defects: circumferential/angular intraosseous 1–4 wall defects, dehiscence defects and fenestration. The main factors that determined the accuracy of CBCT were mainly the size of the defects and the presence of artefacts, while intraoral radiography appeared to be more influenced by the location and the type of defect. The two-dimensionality of 2D radiography resulted in the main limitation in the detection of peri-implant defects, especially in the analysis of dehiscence and fenestration defects. In the study by Vadiati Saberi et al., in which CBCT was compared with periapical radiography and orthopantomography in the detection of defects, CBCT showed the highest values of sensitivity and specificity in detecting all types of defects, while periapical radiography was considered unable in revealing fenestration and dehiscence defects [1]. Interestingly, all fenestrations were properly diagnosed with orthopantomography; one explanation for this result seems to be that, unlike periapical radiography, orthopantomography refers to a type of radiography in which the incident beam is projected at an acute horizontal angle in relation to the object and the image receiver, capable of projecting some parts in the vestibular faces of the implants [1]. On the other hand, with regard to the interproximal faces of the implants, Steiger Roney et al. reported that intraoral radiography was significantly more accurate than CBCT, leading to an overestimation of the defect width, caused by particularly prominent artefacts in this area in relation to others [6].

In regard to detecting circumferential intraosseous defects, peri-apical radiography demonstrated greater diagnostic accuracy than in detecting dehiscence and fenestration defects; however, CBCT has demonstrated to be the most accurate technique, capable of providing additional information on the location and precise morphology of the defects, as the lower effectiveness of periapical radiography in detecting dehiscence defects and fenestrations was mainly due to its two-dimensional nature, unable to show the buccal and lingual parts of the implants.

Schwindling et al. and Hilgelfeld et al. reported that the precision and reliability were comparable between intraoral radiography and CBCT in relation to the detection of 1–4 wall intraosseous circumferential defects, that is, the separation of samples with and without defects.

Regarding the more complex task of defect classification, that is, the recognition of defect geometry, significant differences were detected between the techniques, with CBCT demonstrating significantly higher sensitivity and specificity values than intraoral radiography [5, 13], as peri-apical images cannot provide volumetric information about the status of defects, which is important in monitoring progression or resolution after treatment [8]; thus, when comparing conventional radiographs to CBCT, additional information can be extracted from CBCT [12]. This way, the use of CBCT has been suggested and encouraged by numerous studies as the modality of choice for assessing bone defects to overcome the limitations of 2D radiography, as it provides improved accuracy, therefore, enabling an early application of clinical therapies to avoid additional bone loss. [18, 19]

### Influence of artefacts on peri-implant bone measurements

The main limitation of CBCT is the presence of metallic artefacts, that is, image flaws unrelated to the digitized object caused by metals such as dental implants and amalgam restorations, or to a lesser extent, by root canal filling materials in endodontic treatments [9]. Several authors have reported peri-implant measurements influenced by image artefacts caused by implants [1, 6, 8, 9, 12, 16], as metal artefacts are frequently found in CBCT images, and in fact, it is important for the diagnosis of implants to know how the effects of “scattering”, “beam hardening” and “blooming” alter the dataset and the visualized image. Scatter artefacts appear as linear radiopacities that radiate from a metallic object and can extend to the width of the field, affecting the visualization of areas, even on the opposite side of the image. The beam hardening artefact appears as a dark zone in the vicinity of the high density object, which can make it difficult to visualize the bone-implant interface, while blooming is an image artefact that creates an overestimation in the size of the scanned object [6, 8]. These image artefacts are well described in the study by Vanderstuyft et al., in which CBCT was evaluated in the analysis of oral peri-implant bone thickness of 44 titanium implants [12], where the oral peri-implant bone was measured clinically by probing and the postoperative CBCTs were taken. Then, the implant was removed, and a new CBCT scan was taken without implant artefacts. Due to an average percentage of blooming (artificial increase in the implant diameter) of 12–15%, the peri-implant buccal bone thickness was underestimated by 0.3 mm immediately adjacent to the flowering area of the implant. An area of uncertainty of approximately 0.45 mm was observed, in which the buccal bone was not always visible, and buccal bone of sufficient thickness to fall out of this uncertain area could always be seen [20].

In the study of Schwindling et al., beam hardening artefacts made it difficult to define the depth of the circumferential defects, while the width measurements substantially agreed with the reference measurements [13], in line with Kamburoglu et al*.*, who found a higher mean deviation from defect status for depth than for width measurements [8]. The explanation for this phenomenon seems to be that, in most cases, the deepest area of the defect was in the direct vicinity of the implant, and in these areas, more prominent artefacts were found than in others [13], as stated by Eskandarloo et al., who found that when a bone defect is adjacent to a dental implant, a radiolucent area is created, making it difficult to accurately detect the defect [17].

The distinction between image artefacts and peri-implant bone loss has often proven to be a challenge, especially since they can be confused with one another, leading to false positives or false negatives. Albeit L.R. Pinheiro et al*.* observed that CBCT images compatible with bone defects were characterized by hypodense and irregular zones, with small hyperdense areas (remnants of trabecular bone), while images produced by metal artefacts were more regular and completely hypodense, suggesting that this small difference can be useful in establishing correct diagnoses [15].

Kamburoglu et al*.* investigated the effect of artefact reduction software on the ability to accurately detect mechanically created peri-implant and periodontal defects from CBCT images and found no difference in the use of artefact reduction software but reported that peri-implant defects were detected with greater difficulty than periodontal defects [9].

However, the results described cannot be generalized, as CBCT scanners from different manufacturers with different spatial resolutions (voxel sizes), fields of view (FOV), patient positioning systems or scanning durations are in use worldwide, which will influence the quality and interpretability of the scans [6].

### Impact of acquisition parameters on peri-implant bone measurements

Several factors in relation to image quality control, exposure to reduced artefacts (for example, mA and kVP) and technical acquisition parameters may have a direct impact on detecting relatively small deficiencies in the implant region, and the size of the field of view (FOV), thickness of the voxel, and the number of acquisition frames can directly influence the image quality of the CBCT scan [15].

FOV is the term used to refer to the scanning volume of a specific CBCT unit and is determined by the size and shape of the detector, beam projection geometry and beam collimation, which limits radiation exposure to a specific region of interest [8]. A “voxel” describes the smallest distinguishable box-like part of a three-dimensional image [8]. In CBCT images, voxels are isotropic, and images can be built on any plane with high fidelity. The availability of different FOVs allows one to select the most appropriate FOV for a specific application, as larger FOVs result in higher effective radiation doses; as a rule, smaller FOVs are recommended for imaging a quadrant or single tooth [8]. Kamburoglu et al*.* and Pinheiro et al*.* tested the influence of different sizes of FOV in the detection of peri-implant bone lesions, and they did not find significant differences in the detection rates; however, the results in the study by Kamburoglu et al.are confusing, since the size of the voxel varies between protocols [8, 15].

According to Pinheiro et al. [15], the proper selection of the CBCT acquisition protocols in the postoperative evaluation of the peri-implant bone is a balance between ideal image quality and an acceptable dose of effective radiation [15].

There is evidence in the field of endodontics and periodontology that acceptable image quality can be achieved with reduced kV, mA and exposure time [13]. Schwindling et al*.* investigated the diagnostic accuracy of a low-dose protocol (LD-CBCT) compared with that of a high-dose protocol (HD-CBCT) in the detection and classification of peri-implant bone lesions [13] and found that a 5 × 5.5 cm FOV, 85 kV, 12 bits and 360° rotation were commonly used by the two protocols, while the differences included in an increase in the exposure time from 2.2 to 14.2 s, an increase from 384 acquisition frames to 767, an increase in passage from 30 to 421 mGycm, and a decrease in the size of the voxel from 160 to 80 μm. These results showed that the performance of HD-CBCT in the detection and classification of defects was superior to that of the LD-CBCT protocol, although not significantly, and that the increase in the radiation dose of the high-dose protocol was not justified, as it was 14 times higher than that of the low-dose protocol [13]. While Schwindling et al. did not change the kVp between the two protocols, L.R. Pinheiro et al*.* conducted a study in which the accuracy of CBCT was compared between 75 and 90 kVp protocols in the detection of circumferential peri-implant defects and found that CBCT images at 90 kVp were associated with a significantly higher rate of detection of chemically simulated bone defects than 75 kVp CBCT [7].

In the study by Eskandarloo et al., in which three CBCT systems for detecting peri-implant fenestration were compared, the CBCT NewTom 3G, which was associated with a higher KVp, showed the highest diagnostic accuracy in detecting peri-implant fenestration, although no significant differences were found between the three CBCT systems [17].

## Conclusion

CBCT images can be used with high diagnostic precision in the detection of peri-implant bone defects, with an average discrepancy of less than 1 mm from the actual size of the defect. CBCT provides useful information about bone in all dimensions around the implants with varying accuracy depending on several factors.

The presence of artefacts in the peri-implant area was the most frequent limitation of CBCT in the detection and measurement of bone defects, resulting in an underestimation or overestimation of the size, depending mainly on the implant material and the sample orientation.

Larger peri-implant defects showed greater detection accuracy, while small defects (less than 1 mm) were more difficult to detect.

Adjusting the acquisition parameters such as FOV, acquisition time and voxel size does not seem to significantly affect the image quality in detecting bone loss, while kVp does. However, the results found on the adjustment of the acquisition parameters are difficult to interpret given that the variables are innumerable. More standardized studies should address this issue.

Intraoral radiography can be recommended as an initial method of diagnosis due to its low cost and low dose of administered radiation, in addition to its comparability to CBCT with regard to mesial and distal peri-implant bone analysis. However, it was unable to show the buccal and oral aspects of the implants and was, therefore, unable to diagnose bone defects such as fenestrations and dehiscence.

In general, CBCT can be considered a useful and reliable tool for the diagnosis of peri-implant bone loss. However, special attention should be given to the radiation dose administered. Further studies comparing different CBCT machines in the diagnosis of peri-implant bone loss should become available, as the market offers a wide variety.
